# The effects of maternal anxiety during pregnancy on *IGF2*/*H19* methylation in cord blood

**DOI:** 10.1038/tp.2016.32

**Published:** 2016-03-29

**Authors:** T Mansell, B Novakovic, B Meyer, P Rzehak, P Vuillermin, A-L Ponsonby, F Collier, D Burgner, R Saffery, J Ryan, BIS investigator team, Peter Vuillermin, Anne-Louise Ponsonby, John B Carlin, Katie J Allen, Mimi L Tang, Richard Saffery, Sarath Ranganathan, David Burgner, Terry Dwyer, Kim Jachno, Peter Sly

**Affiliations:** 1 Cancer & Disease Epigenetics, Murdoch Childrens Research Institute, Royal Childrens Hospital, Parkville, VIC, Australia; 2 Department of Paediatrics, The University of Melbourne, Parkville, VIC, Australia; 3 Ludwig-Maximilians-University of Munich, Division of Metabolic and Nutritional Medicine, Dr. von Hauner Children's Hospital, University of Munich Medical Centre, Munich, Germany; 4 Child Health Research Unit, Barwon Health, Geelong, VIC, Australia; 5 School of Medicine, Deakin University, Geelong, VIC, Australia; 6 Inserm U1061, Hopital La Colombiere, Universite Montpellier, Montpellier, France; 7 Department of Epidemiology and Preventative Medicine, School of Public Health and Preventative Medicine, Monash University, Prahran, VIC, Australia

## Abstract

Compelling evidence suggests that maternal mental health in pregnancy can influence fetal development. The imprinted genes, insulin-like growth factor 2 (*IGF2*) and *H19,* are involved in fetal growth and each is regulated by DNA methylation. This study aimed to determine the association between maternal mental well-being during pregnancy and differentially methylated regions (DMRs) of *IGF2* (DMR0) and the *IGF2/H19* imprinting control region (ICR) in newborn offspring. Maternal depression, anxiety and perceived stress were assessed at 28 weeks of pregnancy in the Barwon Infant Study (*n*=576). DNA methylation was measured in purified cord blood mononuclear cells using the Sequenom MassArray Platform. Maternal anxiety was associated with a decrease in average ICR methylation (Δ=−2.23% 95% CI=−3.68 to −0.77%), and across all six of the individual CpG units in anxious compared with non-anxious groups. Birth weight and sex modified the association between prenatal anxiety and infant methylation. When stratified into lower (⩽3530 g) and higher (>3530 g) birth weight groups using the median birth weight, there was a stronger association between anxiety and ICR methylation in the lower birth weight group (Δ=−3.89% 95% CI=−6.06 to −1.72%), with no association in the higher birth weight group. When stratified by infant sex, there was a stronger association in female infants (Δ=−3.70% 95% CI=−5.90 to −1.51%) and no association in males. All the linear regression models were adjusted for maternal age, smoking and folate intake. These findings show that maternal anxiety in pregnancy is associated with decreased *IGF2*/*H19* ICR DNA methylation in progeny at birth, particularly in female, low birth weight neonates. ICR methylation may help link poor maternal mental health and adverse birth outcomes, but further investigation is needed.

## Introduction

Maternal mental health during pregnancy has been linked with poor fetal growth and lower birth weight,^1, 2, 3^ as well as adverse health outcomes in childhood, including neurodevelopmental delay,^4, 5^ attention problems and depressive symptoms.^6, 7, 8^ An adverse *in utero* environment is speculated to lead to ‘programmed' changes in the fetus during development,^9^ and epigenetic processes including DNA methylation are thought to have an important role.^9, 10^ Indeed, increasing evidence has shown that maternal mental well-being during pregnancy, influenced by factors such as stress, anxiety and depression, can influence DNA methylation levels of the glucocorticoid receptor gene in the neonate.^11^ Several recent studies also provide preliminary evidence that maternal mental health during pregnancy can result in differential methylation levels in imprinted genes in the offspring.^12, 13, 14^ Infants born to women with severe depression during pregnancy (*n*=32) had higher cord blood methylation of the *MEG3* imprinted gene than infants born to 271 non-depressed women.^12^ Similarly, cord blood methylation of another imprinted gene, *MEST*, was positively correlated with maternal perceived stress during pregnancy in a recent study of 79 mother–infant pairs.^14^

Imprinted genes are involved in a number of pathways crucial for fetal growth,^15^ and alleles for imprinted genes are expressed according to parental origin. DNA methylation levels differ between parental alleles at imprinted genes in association with differential gene expression.^16^ Of particular interest are the reciprocally imprinted *IGF2*/*H19* genes. *IGF2* encodes insulin-like growth factor 2 (IGF2), the major growth hormone during fetal life,^17^ expressed specifically from the paternal allele, whereas the downstream *H19* gene is specifically expressed from the maternal allele.^18^ The imprinting pattern is regulated by methylation at the imprinted control region (ICR) and is associated with several differentially methylated regions (DMRs) in the IGF2 and H19 gene promoters.^19^ Of note, methylation of *IGF2* DMR0 and DMR2 has been linked with infant birth weight.^20^ Offspring IGF2 and H19 methylation has been shown to be influenced by *in utero* exposures. For example, lower *IGF2* DMR0 methylation has been reported in the whole blood of adults born to mothers who experienced famine during the Dutch Hunger Winter,^21^ whereas elevated DMR0 methylation^22^ and lower H19 promotor DMR^23^ has been found in infants exposed to folic acid supplementation during pregnancy. Higher *H19* promoter DMR methylation has been found in association with macronutrient intake in a study that combined measurements from buccal, cord blood, umbilical vein endothelium and placenta and granulocytes cells.^23^ Maternal smoking during pregnancy has also been shown to influence newborn *IGF2* DMR0 and *H19* promotor DMR methylation.^13, 24^

Two recent studies have found evidence of associations between maternal mental health and methylation of *H19* and *IGF2*. Chen *et al.*^25^ reported increased methylation of the ICR located upstream of *H19* in cord blood and placenta tissue of infants born to mothers with high self-reported stress and anxiety, whereas Vangeel *et al.*^26^ found decreased methylation of *IGF2* DMR0 in association with maternal anxiety. These findings based on relatively small sample sizes (*n<*100) have not yet been replicated. The associations between maternal mental well-being and *IGF2*/*H19* methylation may differ across ethnic groups,^13^ and could vary depending on the infant's gender and birth weight.^12, 14^ Given the multitude of factors known to influence DNA methylation both temporally and spatially, ascertaining the effects of any exposure *in utero* on infant outcome are best addressed using large, well-characterized population-based birth cohort studies with robust measurements of exposure and biospecimens collected at birth.

In the current study, we assessed whether maternal mental health during pregnancy (depression, anxiety and perceived stress) was associated with cord blood methylation, focusing on the *IGF2*/*H19 ICR*, one of the key imprinted loci, and *IGF2* DMR0. These two regions are of particular interest given that they have been the most widely studied in previous studies and have been shown to be differentially methylated in response to other *in utero* exposures.^21, 23, 25, 27, 28, 29, 30^ Second, we aimed to determine whether other key factors such as maternal smoking during pregnancy, infant gender and birth weight, influenced this association.

## Materials and methods

### Study population

The prospective Barwon Infant Study^31^ is a population-derived birth cohort of mother–infant dyads (*n*=1074). The study was established in 2010 to investigate the developmental and early-life origins of non-communicable disease.^32^ The study protocol was approved by the Barwon Health Human Research Ethics Committee, and the participants provided written informed consent.

Women were recruited from two hospitals in the Geelong region of Victoria between 15 and 28 weeks of pregnancy; Geelong Hospital (public) and St John of God Hospital (private). Those invited to participate needed to be living in the Barwon region, an Australian citizen or permanent resident, aged at least 18 years at 28 weeks of pregnancy and able to complete questionnaires and provide informed consent. Women were excluded from participating if they already had another child in the study (with the exception of twins), or had planned to privately store their cord blood. Following birth, neonates were excluded if they were born before 32 weeks gestation, diagnosed with a serious illness at birth or had a genetically determined disease or major congenital malformation. Twins were also excluded from the current analysis.

At 28 weeks gestation, mothers were administered comprehensive questionnaires to gather sociodemographic, clinical and health-related information. This included parental age, education levels and ancestry. Maternal alcohol consumption and tobacco smoking across pregnancy were also recorded, as were pregnancy-related health conditions, such as preeclampsia, hypertension and gestational diabetes (based on standard criteria). At birth, information was gathered from medical records, including the neonate's gender, gestational age and birth weight.

### Maternal mental well-being during pregnancy

Specific aspects of maternal mental well-being during the twenty-eighth week of pregnancy were assessed using two validated and widely used self-report questionnaires. The 10-item Edinburgh Depression Scale (EDS) ^33^ is a questionnaire focused on mood and feelings over the past week.^34^ It provides an assessment of depression and anxiety symptoms, which can be distinguished from one another reliably (reviewed by Matthey *et al.*,^35^ where Cohen's *d* ranged from 0.61 to 1.41 across five different studies). We classified women as having clinically significant levels of depression if they scored 10 or more on the EDS, based on previous reports that this provided optimal sensitivity and specificity.^36^ This cut-off is also the most widely used in the literature. Anxiety symptoms were defined as a score of 5 or more on the EDS anxiety subscale, as previously described.^37, 38^

Maternal psychological stress during pregnancy was assessed with the validated Perceived Stress Scale.^39^ This 14-item questionnaire focuses on perceived stress experienced in the last month, and has been shown to provide a more accurate reflection of the biological impact of psychological stress compared with measuring stressful life events.^40^ The Perceived Stress Scale scores ranged from 0 to 50, with higher scores indicating greater distress.

### Cord blood processing

The umbilical cord blood was collected at delivery and processed within 18 h. Density gradient centrifugation (Lymphoprep, AxisShield, Dundee, UK) was used to isolate mononuclear cells from the whole-blood sample, and cell number and viability assessed by Trypan Blue staining. The cell composition of each cord blood mononuclear cells sample (lymphocyte, monocyte and/or contaminating erythrocytes) was determined by flow cytometry. The cells were stained with antibodies to CD3-FITC, CD4-PE and CD45-PerCP (BD Biosciences, San Jose, CA, USA). To evaluate the relative frequency of lymphocyte, monocyte and any contaminating nucleated erythrocytes, the cell populations were gated on the basis of CD45 positivity and granularity (high side scatter, SSC).

### DNA methylation

The assays were designed using the EpiDesigner software (www.epidesigner.com) to cover the key regions of *IGF2* and *H19* identified in previous studies.^41^ The cleavage patterns were determined using the Bioconductor MassArray package in R (www.bioconductor.org). For *IGF2* DMR0, the primers (forward 5′-TGGATAGGAGATTGAGGAGAAA-3′ reverse 5′-AAACCCCAACAAAAACCACT-3′) amplified a 338 bp DMR of the promoter (hg_18:chr11:2126035–2126372). For the *IGF2*/*H19* ICR, the primers (forward 5′-TATGGGTATTTTTGGAGGTTTTTTT-3′ reverse 5′-AACTTAAATCCCAAACCATAACACT-3′) amplified a 316 bp DMR of the ICR (hg_18:chr11:1977554–1977869). For both the assays, the forward primer contained a balance tag (AGGAAGAGAG) and the reverse primer a T7 tag (5′- CAGTAATACGACTCACTATAGGGAGAAGGCT-3′).

The QIAamp DNA Mini Kit (QIAGEN, Chadstone, Australia) was used to extract DNA. The EZ-96 DNA Methylation-Gold kit (Zymo Research, Irvine, CA, USA) was used for bisulphite conversion of 500 ng of genomic DNA. SEQUENOM MassARRAY (San Diego, CA, USA) and the EpiTyper software (v.1.2; SEQUENOM) were used for the quantification of DNA methylation.^42^ All the samples were amplified and assayed in triplicate. Technical replicates which deviated from the mean by 10% or more were excluded, and the mean of the duplicates was used for subsequent analysis. If two or more of the triplicates deviated from one another, the sample was considered as missing and not used for the subsequent analysis given that it was unreliable. If <70% of the mean values were successfully measured for a given CpG site, or if <70% of CpG sites were successfully measured for a given sample, this was excluded.

### Statistical analysis

The study sample was 576 mother–infant dyads with complete information on the maternal mental well-being during pregnancy and DMR0 and/or ICR methylation data. Compared with the complete BIS population, the participants included in this study did not differ in sociodemographic or health status (data not shown).

Pair-wise correlations, *t*-tests, analysis of variance and chi-squared tests (as appropriate) were used to determine the association between specific maternal mental health measures during pregnancy and characteristics of the mother–infant dyads, as well as the association between these characteristics and DNA methylation. Multiple linear regression models were constructed to examine the association between the maternal mental health and CpG methylation while adjusting for potential confounding factors. Although none of the maternal or infant characteristics (listed in Table 1) were associated with both the exposure and outcome at *P<*0.1, we ensured that their inclusion in the model did not influence the associations found. The cell composition as well as possible batch effects from individual SEQUENOM were also considered as potential confounding factors in the analysis. We also investigated the possibility that the association between maternal mental health and DNA methylation differed across strata of participants. First-order interaction terms were investigated with maternal smoking, infant gender and birth weight (both raw and adjusted birth weight, taking into account gestational age and infant sex^43^) by including a product term in the regression models. If an interaction was found, stratum-specific results are reported. All the statistical analysis was undertaken using Stata 14 IC version (StataCorp, College Station, TX, USA).

## Results

Mothers in the study had a mean age at delivery of 32.1 years (s.d.=4.5) and 46.0% (265/576) were educated to at least a university graduate level. The prevalence of maternal smoking and drinking was 9.2% (53/576) and 11.6% (63/576), respectively. One-hundred and six women reported depressive symptoms during pregnancy (18.4% of 576), and 19.3% (111/576) of women had anxiety symptoms (of which 76 had both anxiety and depressive symptoms). The mean perceived stress scores were 18.9 (s.d.=7.1), and ranged from 2 to 44. The three mental health measures were highly correlated with one another (*P*<0.0001). In terms of the association between maternal mental health and maternal health during pregnancy, anxiety during pregnancy was associated with increased incidence of hypertension (*P*=0.023) and perceived distress was associated with gestational diabetes (*P*=0.010). All mental health measures were associated with a small decrease in gestational age (*P*<0.02 for each) but there was no association between maternal mental health and birth weight (anxious, *P*=0.28; depressed, *P*=0.74; perceived distress, *P*=0.89) or birth weight adjusted for gestational age (anxious, *P*=0.70; depressed, *P*=0.21; perceived stress, *P*=0.36).

The mean and standard deviation of *IGF2* DMR0 and *IGF2*/*H19* ICR DNA methylation levels in the sample as well as the mass spectrometry success rate at individual CpG units and the average level across the regions are shown in Supplementary Table 1. The methylation levels at all the included CpG units were normally distributed. Within the DMR0 and ICR regions, methylation levels at the individual CpG units were correlated with one another (*P<*0.01, data not shown). The characteristics of the participants and average DMR0 and ICR methylation are shown in Table 1. None of the characteristics were significantly associated with average DNA methylation of either region; however, increased DMR0 methylation was observed in infants born to mothers with gestational diabetes (*P*=0.06), compared with those without.

**Figure 1:**
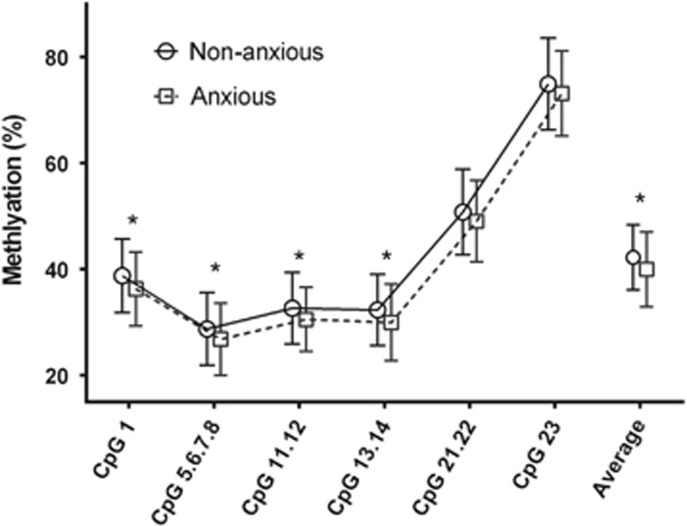
Population distribution of *IGF2*/*H19* ICR DMR methylation levels in infants born to anxious and non-anxious mothers. *P⩽0.016. Error bars are standard deviation. DMR, differentially methylated region; ICR, imprinting control region.

We first investigated whether DMR0 and ICR mean methylation levels differed according to maternal mental health status during pregnancy. In unadjusted analysis, maternal anxiety symptoms were found to be associated with a 2.23% decrease in average ICR methylation across the region (95% CI=0.77 to 3.68% *P*=0.003). At individual CpG units, a number of group differences were also observed, in each case with decreased methylation levels in the anxious group: 2.48% difference at CpG 1 (95% CI=0.84 to 4.12% *P*=0.003); 1.94% at CpG 5.6.7.8 (95% CI=0.36 to 3.52% *P*=0.02); 2.13% at CpG 11.12 (95% CI=0.60 to 3.66% *P*=0.01); and 2.38% at CpG 13.14 (95% CI=0.80 to 3.96% *P*=0.003; Figure 1). At the other CpG units, 21.22 and 23, there was a trend of decreased methylation in the anxiety group (CpG 21.22: Δ=−1.70%, 95% CI=−3.59 to 0.19%, *P*=0.078; CpG 23: Δ=−1.79%, 95% CI=−3.90 to 0.32%, *P*=0.096). Maternal anxiety was also associated with a 1.83% decrease at DMR0 CpG 3 specifically, (95% CI=0.28 to 3.38%, *P*=0.02), but no association was observed between anxiety and average DMR0 methylation. There were similar associations with ICR methylation found for maternal depression and perceived stress (particularly at CpG 1, CpG 11.12 and CpG 13.14), but these were weaker and less consistent than with anxiety (Supplementary Table 2). In linear regression models adjusted for key covariates previously reported to influence DNA methylation levels and possibly associated with maternal mental health, for example, maternal age, smoking and folate, infant gender and birth weight (raw and adjusted *z*-scores), these associations persisted (Supplementary Table 3).

There was also some evidence of effect modification in the association between anxiety and ICR methylation according to birth weight (*P* for interaction 0.03 for average methylation, 0.10 for CpG 1, 0.05 for CpG 5.6.7.8, 0.08 for CpG 13.14, 0.02 for CpG 21.22) and infant sex (average methylation, *P* for interaction 0.07). To investigate how the association between maternal anxiety during pregnancy and ICR methylation varied according to birth weight, we repeated the analysis in groups stratified by birth weight. There were too few infants in the clinically defined low (<2500 g) or high birth weight (>4500 g) groups, so we created a binary group using the median (3530 g) birth weight. The results, as shown in Table 2, indicate that maternal anxiety during pregnancy was strongly associated with lower ICR methylation in the lower birth weight group (⩽3530 g) only. The maternal anxiety was associated with a 3.89% decrease in average ICR methylation (95% CI=1.72 to 6.06%, *P*<0.001), however, no such association was observed in the higher birth weight group (Δ=−0.59%, 95% CI=−2.54 to 1.37%, *P*=0.55; Supplementary Figure 1). When the analysis was repeated with birth weight adjusted for gestational age and infant sex, findings were consistent with those using raw birth weight.

Given our previous observation that infant sex also modified the association between anxiety and DNA methylation, sex-stratified analysis was undertaken. The results showed a female-specific association between anxiety and decreased ICR methylation (Supplementary Table 4), which corresponded to a mean 3.70% decrease in average ICR methylation (95% CI=1.51 to 5.90%, *P*=0.001), and varied from 2.71% to 3.36% at the individual CpG units. No associations were found in males.

**Figure 2:**
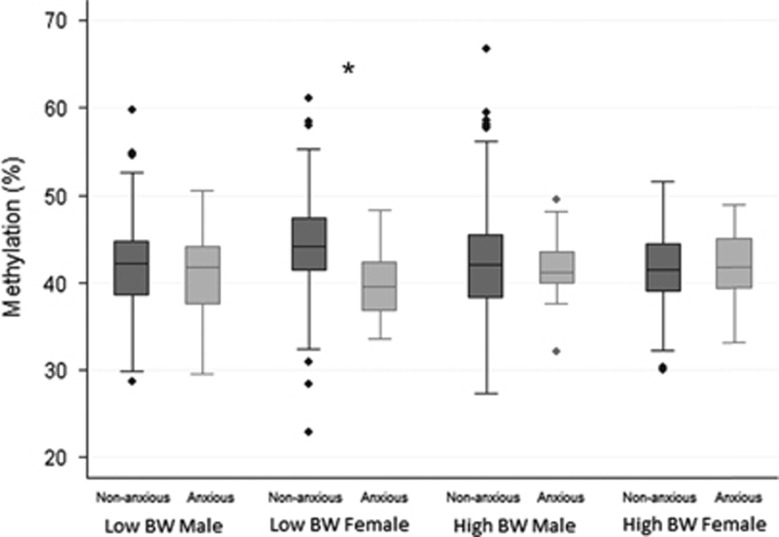
Population distribution of *IGF2*/*H19* ICR DMR methylation levels in non-anxious and anxious groups, stratified by both infant sex and birth weight. *P=0.0001. BW, birth weight; DMR, differentially methylated region; ICR, imprinting control region.

As female infants were more likely to be in the lower birth weight group than males (57 versus 44%), we further investigated how the association between anxiety and DNA methylation differed across groups defined by both gender and birth weight (Figure 2). The strongest association between maternal anxiety during pregnancy and infant ICR methylation was observed for female infants in the lower birth weight group, with a 3.86% decrease in methylation levels. For all the results, sensitivity analysis confirmed that the removal of outliers (represented by individual points in Figure 2 and Supplementary Figure 1) did not alter the findings.

## Discussion

This study utilized data gathered from a large longitudinal birth cohort to investigate the association between specific maternal mental health measures and infant methylation of the *IGF2*/*H19* ICR and *IGF2* DMR0. We report strong and consistent evidence of an association between maternal anxiety and decreased *IGF2*/*H19* ICR methylation at the major CpG sites across the region investigated and these associations persisted when accounting for a range of covariates. Similar, but weaker, associations were also observed with maternal depression and perceived stress during pregnancy. The maternal mental well-being is one of a number of environmental factors which have now been shown to influence *IGF2* and *H19* methylation, along with smoking,^24^ antidepressant use^13^ and famine.^21^ Only two smaller studies (*n<*100) have previously considered the association between maternal anxiety and *IGF2/H19* methylation, and their findings have not yet been replicated.^25, 26^

Some recent studies have investigated possible associations between maternal mental health and infant *IGF2* and *H19* methylation in cord blood. Of these, one study has examined the relationship between anxiety and methylation of ICR CpGs 11 to 17, as measured here. This small study^25^ contrasted with our own findings and reported increased methylation in association with maternal anxiety (measured by the State-Trait Anxiety Inventory) and perceived stress (Perceived Stress Scale) in cord blood but no association was found in matched placental tissue. However, this study considered only 49 mother–infant dyads and the association between anxiety and methylation did not persist when considering anxiety disorders diagnosed through clinical interviews. Two other studies using cord blood have investigated the association between anxiety, *IGF2* and ICR methylation, but they assayed slightly different gene regions compared with our own study. One small study (*n*=80), focussing on maternal anxiety measured using the Pregnancy-Related Anxiety Questionnaire, found a decrease in DMR0 methylation at a CpG site outside our assay.^26^ The other larger study (*n*=508) considered self-reported anxiety, but reported no association at either a region upstream of *IGF2* exon 3 or the ICR.^12^ These two studies also investigated depression, one finding decreased methylation at a CpG site outside our assayed region^26^ and the other finding no association with methylation of either region, though an association was reported at another imprinted gene, *MEG3*.^12^ Two further studies using cord blood samples have investigated *IGF2* and *H19*, albeit at different regions to our study, and have not considered anxiety. A pregnancy cohort partially enriched for smokers (*n*=436) found no association between self-reported depression and DNA methylation levels.^13^ Birth weight, but not infant sex was considered as a covariate in their analysis. The other small study (*n*=79) investigated potential associations between perceived stress (Perceived Stress Scale) and methylation of nine imprinted gene DMRs, including *IGF2* and *H19*.^14^ The only association observed was increased methylation at the *MEST* imprinted gene.

We found that the association between maternal anxiety and decreased ICR methylation differed according to birth weight and infant sex, being strongest in low birth weight infants and girls. This is notable as maternal anxiety and decreased infant *IGF2*/*H19* ICR methylation have both previously been linked with lower birth weight,^20, 44^ though we did not observe such an association in this study. Gender-specific associations have also been reported previously, with stronger effects of adverse maternal mental health reported in females for methylation of *IGF2*, *MEG3* (ref. 12) and *MEST*.^14^

Our finding of decreased ICR methylation in neonates born to mothers with anxiety could help to explain the previously reported link between poor maternal mental health and adverse birth outcomes, including low birth weight and neural tube defects.^1, 45, 46^ The methylation of the *IGF2*/*H19* ICR on the maternal allele prevents the binding of transcriptional repressor CTCF, which allows access to the downstream enhancer region of *IGF2.* This promotes *IGF2* expression to produce insulin-like growth factor 2.^47^ The IGF2 protein binds to insulin-like growth factor 1 receptor (encoded by *IGF1R*), a kinase activator that promotes cell survival and proliferation signals^48^ and consequently promotes fetal growth. In contrast, when CTCF is bound, this allows *H19* to be expressed to produce miR-675, which has been shown to supress growth during gestation and is linked to downregulation of *IGF1R*.^49^ Increased *IGF2* DRM0 methylation has also been linked to decreased *IGF2* mRNA expression levels.^50^ In turn, this has been associated with lower birth weight^20^ and other health problems such as neural tube defects.^50^

A strength of our study is the large well-characterized cohort, which has allowed us to consider a range of covariates in the analysis, including several that have previously been associated with ICR methylation. However, we found no independent association between any covariates and DNA methylation levels of DMR0 and ICR. *IGF2* and *H19* are known to have a role in regulating fetal growth through insulin-like growth factor 2 (refs 48, 49) and an earlier study has reported a weak positive correlation between birth weight and *IGF2*/*H19* ICR methylation in twins.^41^ Decreased *IGF2* methylation has also been found in clinically low birth weight infants compared with those of normal birth weight.^12^ As only six infants in our study could be considered clinically low birth weight infants (<2500 g), this may help explain the lack of independent association between birth weight and *IGF2* methylation demonstrated here. Another limitation of our study is the single measurement of maternal mental health made at 28 weeks gestation, which means we cannot generalize these findings to mental health status at the other stages of pregnancy. However, a prior study that collected multiple measurements throughout pregnancy reported that second trimester scores for depression, anxiety and perceived stress were highly correlated with scores at other time points.^51^ In addition, a study investigating the association between maternal mental health during pregnancy and neonate methylation of another gene involved in stress signalling found that the second trimester mental health scores were more strongly associated with neonate methylation than the first or third trimester scores.^52^ We considered three distinct mental health exposures, and found similar, though weaker, associations with perceived stress as reported for anxiety. Increased perceived stress was also associated with decreased neonate ICR methylation, particularly at CpGs 1, 11.12 and 13.14. Finally, the difference in ICR methylation between anxious and non-anxious groups found in this study is relatively small. It is possible that the accumulation of small differences in methylation like this across multiple genes within the same biological pathway or in the broader epigenome might be what leads to significant increases of risk of disease in later life, and other studies investigating the effects of maternal mental well-being on neonate methylation have reported similarly small effect sizes,^12, 14, 52, 53, 54, 55^ supporting our own findings. On the basis of previous studies,^47, 56^ decreased ICR methylation would be predicted to lead to reduced CTCF binding and consequently reduced *IGF2* expression, but the potential long-term impact on the neonate's health and development has yet to be established. Although *IGF2*/*H19* ICR methylation is associated with fetal growth,^57^ it is unknown how persistent these changes are into later life and whether they are transient during development or are established as disrupted methylation patterns that remain over time.

## Conclusion

This study provides consistent evidence in a large, well-characterized population-based birth cohort of a prospective association between maternal anxiety during pregnancy and reduced infant *IGF2*/*H19* ICR cord blood methylation. This association persisted when accounting for a range of lifestyle and birth-related covariates, but appeared to be gender-specific and stronger for lower birth weight infants. Though we provide evidence for a potential link between measures of maternal well-being during pregnancy and adverse birth outcomes, possibly through differential *IGF2*/*H19* ICR methylation observed at birth, this has yet to be replicated. Further work is required to characterize the relationships between maternal anxiety, *IGF2*/*H19* ICR methylation, birth outcomes and subsequent long-term offspring health.

## Figures and Tables

**Table 1:** Average methylation of *IGF2* DMR0 and *IGF2*/*H19* ICR DMR according to participant characteristics

*Characteristics*	*Average IGF2 DMR0 methylation*		*Average IGF2/H19 ICR methylation*	
	r	P	r	P
Maternal age (years)	0.021	0.64	0.025	0.60
				
	*Mean % (55)*	P	*Mean (55)*	P	
Maternal education		0.26		0.17	
⩽Year 10	52.55 (5.12)		44.20 (5.50)		
Year 11 or 12	51.67 (6.43)		41.25 (6.44)		
University graduate or above	52.86 (6.13)		41.92 (6.71)		
Other	52.68 (5.74)		41.58 (5.04)		
				
Paternal education		0.96		0.59	
⩽Year 10	52.22 (6.21)		42.55 (5.98)		
Year 11 or 12	52.63 (6.00)		42.07 (6.18)		
University graduate or above	52.36 (6.50)		41.36 (6.45)		
Other	52.34 (5.54)		41.33 (7.23)		
				
Infant ancestry		0.15		0.21	
British	52.04 (6.20)		42.18 (5.58)		
Mixed	52.66 (6.18)		41.28 (6.76)		
Non-British	53.59 (5.32)		42.60 (7.65)		
					
*Smoking during pregnancy*					
No	52.49 (6.12)	0.69	41.91 (6.55)	0.39	
Yes	52.84 (5.52)		41.05 (4.54)		
					
*Alcohol during pregnancy*					
No	52.51 (6.15)	0.80	41.75 (6.38)	0.63	
Yes	52.29 (5.86)		42.16 (6.07)		
					
*Gestational diabetes*					
No	52.41 (6.10)	0.06	41.85 (6.30)	0.28	
Yes	54.74 (5.90)		40.29 (7.92)		
					
*Hypertension*					
No	52.64 (5.88)	0.13	41.81 (6.31)	0.74	
Yes	51.22 (8.06)		41.48 (7.16)		
					
*Preeclampsia*					
No	52.55 (6.00)	0.48	41.83 (6.24)	0.25	
Yes	51.34 (9.39)		39.58 (11.04)		
					
*Child sex*					
Male	52.20 (5.85)	0.20	41.74 (6.26)	0.68	
Female	52.87 (6.38)		41.98 (6.60)		
				
	r	P	r	P	
Child gestational age (days)	−0.020	0.67	0.060	0.24	
Child birth weight (g)	−0.011	0.80	−0.032	0.49	
Antenatal folate (nmol l^−1^)	0.019	0.67	0.031	0.51	

Abbreviations: DMR, differentially methylated region; ICR, imprinting control region.

**Table 2:** Difference in DNA methylation levels between neonates born to anxious versus non-anxious mothers, stratified by birth weight

*CpG unit*	*Lower birth weight (⩽3530 g)*			*Higher birth weight (>3530 g)*		
	*Δ*	*95% CI*	P	*Δ*	*95% CI*	P
*IGF2 DMR0*						
3	−1.66%	−3.91 to 0.59%	0.15	−1.96%	−4.10 to 0.18%	0.072
4	0.30%	−2.23 to 2.84%	0.81	0.40%	−2.89 to 3.70%	0.81
6.7	−0.88%	−2.78 to 1.01%	0.36	0.79%	−0.86 to 2.14%	0.34
Average	−1.00%	−2.71 to 0.70%	0.25	−0.09%	−2.04 to 1.85%	0.92
						
*IGF2*/*H19* ICR						
1	−4.33%	−6.83 to −1.84%	0.0007	−0.70%	−2.83 to 1.44%	0.52
5.6.7.8	−3.40%	−5.71 to −1.10%	0.0040	−0.45%	−2.63 to 1.73%	0.68
11.12	−3.82%	−6.10 to −1.55%	0.0011	−0.47%	−2.53 to 1.63%	0.66
13.14	−4.57%	−6.99 to −2.14%	0.0003	−0.25%	−2.31 to 1.80%	0.81
21.22	−3.76%	−6.49 to −1.04%	0.0071	0.27%	−2.35 to 2.88%	0.84
23	−2.21%	−5.19 to 0.78%	0.15	−1.49%	−4.47 to 1.49%	0.33
Average	−3.89%	−6.06 to −1.72%	0.0005	−0.59%	−2.54 to 1.37%	0.55

Abbreviations: DMR, differentially methylated region; ICR, imprinting control region.
