# Supplementary material for: The effects of maternal anxiety during pregnancy on IGF2/H19 methylation in cord blood
Source: Transl Psychiatry. 2016 Mar 29;6(3):e765–. doi: 10.1038/tp.2016.32 (PMC4872456; doi:10.1038/tp.2016.32)
Supplement: Supplementary Information [file tp201632x1.doc]

*

*


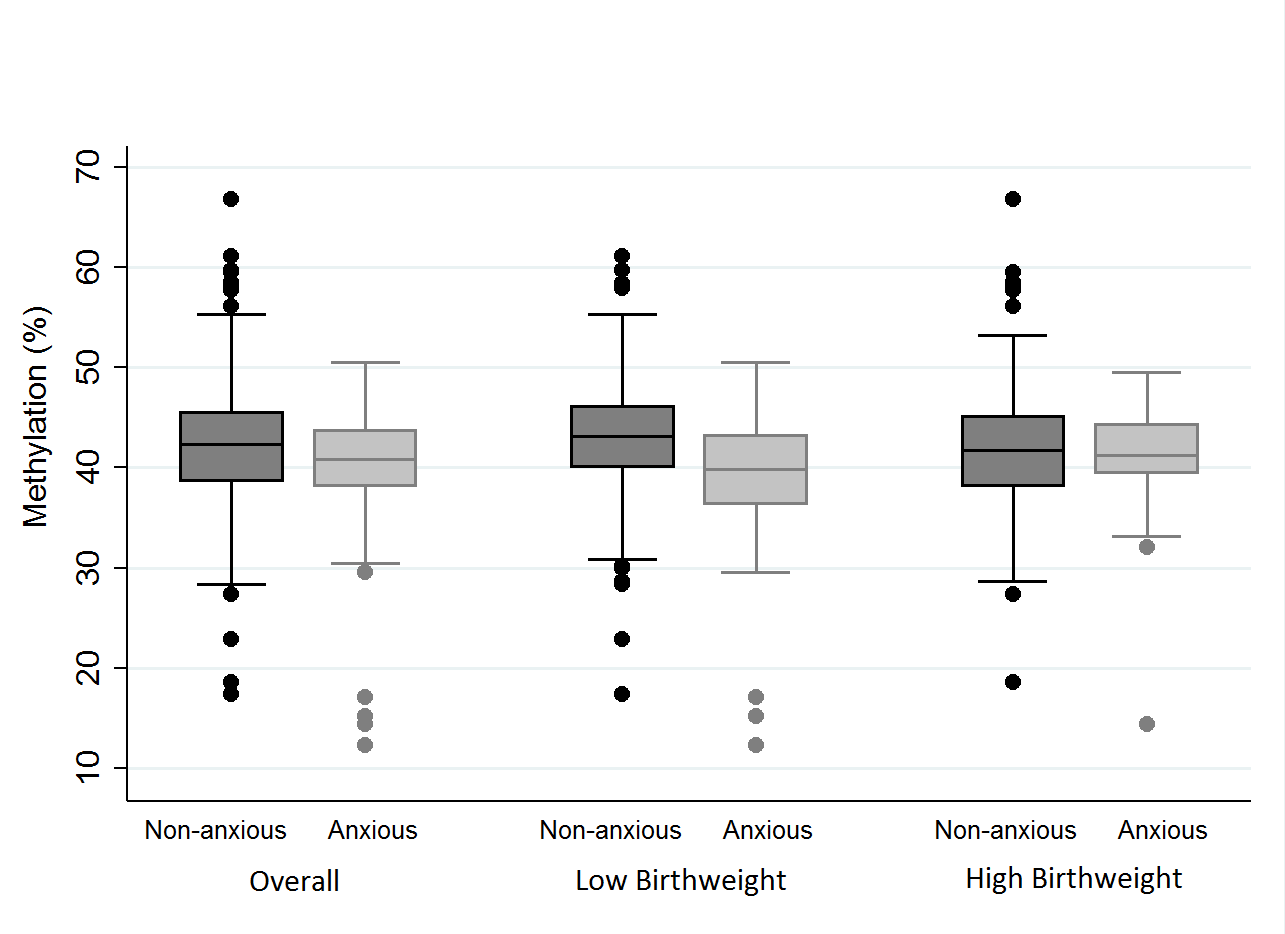


**Supplementary Figure 1.** Population distribution of *IGF2*/*H19* ICR DMR methylation levels in non-anxious vs anxious groups, stratified by infant birthweight. * = p<0.05.

**Table 1. Information on individual CpG units assayed and analysed in this study.**

| **Amplicon** | **CpG site** | **Included** | **Reason for exclusion from analysis** | **Success^b^ (%)** | **Mean methylation** | **SD** |
| --- | --- | --- | --- | --- | --- | --- |
| *IGF2* DMR0 | CpG 1 |  | SNP rs3741208^a^ |  |  |  |
| (DMR0) | CpG 2 |  | SNP rs3741209^a^ |  |  |  |
|  | CpG 3 | Yes |  | 94.0 % | 56.12% | 7.18 |
|  | CpG 4 | Yes |  | 94.4 % | 61.94% | 9.44 |
|  | CpG 5 |  | Low success rate | < 70 % |  |  |
|  | CpG 6.7 | Yes |  | 93.7 % | 39.46% | 5.72 |
|  | CpG 8 |  | Silent peak |  |  |  |
|  | CpG_mean | Yes |  |  | 52.55% | 6.02 |
|  |  |  |  |  |  |  |
| *IGF2/H19* ICR | CpG 1 | Yes |  | 79.8 % | 38.14% | 6.58 |
| (ICR) | CpG 2 |  | Overlapping peak |  |  |  |
|  | CpG 3 |  | Silent peak |  |  |  |
|  | CpG 4 |  | Silent peak |  |  |  |
|  | CpG 5.6.7 | Yes |  | 83.0 % | 28.46% | 6.56 |
|  | CpG 9 |  | Overlapping peak |  |  |  |
|  | CpG 10 |  | Low success rate | < 70 % |  |  |
|  | CpG 11.12 | Yes |  | 82.0 % | 32.14% | 6.40 |
|  | CpG 13.14 | Yes |  | 82.1 % | 31.93% | 6.53 |
|  | CpG 15.16.17 |  | Overlapping peak |  |  |  |
|  | CpG 18 |  | Overlapping peak |  |  |  |
|  | CpG 19 |  | Overlapping peak |  |  |  |
|  | CpG 20 |  | Overlapping peak |  |  |  |
|  | CpG 21.22 | Yes |  | 81.6 % | 50.39% | 7.63 |
|  | CpG 23 | Yes |  | 72.7 % | 74.08% | 8.53 |
|  | CpG_mean | Yes |  |  | 41.79% | 5.94 |
|  |  |  |  |  |  |  |

^a^The methylation distribution suggested that this CpG unit was influenced by genetic variation (SNP), and this is supported by published findings (57)

^b^Of individual samples where at least 2 of the triplicate values remained after quality control. Samples where less than 2 of the replicates were successfully genotyped were excluded.

Silent peak and Overlapping peak: according to the SEQUENOM Mass Spectrometry

**Supplementary Table 2.** Associations of neonate cord blood methylation levels with maternal depression (depressed versus non-depressed mothers) and perceived stress (continuous PSS score).

|  | **Depression** | | **Perceived stress** | |
| --- | --- | --- | --- | --- |
| **CpG unit** | **∆** | **p** | **r** | **p** |
| ***IGF2* DMR0** |  |  |  |  |
| 3 | -0.86% | 0.29 | -0.0003 | 0.99 |
| 4 | -1.00% | 0.33 | -0.038 | 0.38 |
| 6.7 | -0.71% | 0.27 | 0.055 | 0.20 |
| Average | -0.86% | 0.19 | -0.0031 | 0.94 |
|  |  |  |  |  |
| ***IGF2*/*H19* ICR** |  |  |  |  |
| 1 | -1.48% | 0.082 | -0.10 | 0.028 |
| 5.6.7.8 | -1.21% | 0.14 | -0.073 | 0.11 |
| 11.12 | -1.36% | 0.084 | -0.096 | 0.035 |
| 13.14 | -1.46% | 0.075 | -0.10 | 0.027 |
| 21.22 | -<0.001% | 0.99 | -0.043 | 0.35 |
| 23 | -0.26% | 0.81 | -0.066 | 0.18 |
| Average | -0.81% | 0.28 | -0.072 | 0.11 |

**Supplementary Table 3.** Adjusted linear regression model of the prospective association between maternal anxiety during pregnancy and average DNA methylation.

| **Variables** | **Average** ***IGF2*/*H19* ICR methylation** | | |
| --- | --- | --- | --- |
|  | β | SE | p |
| **Maternal anxiety during pregnancy** | -.0204 | .0078 | 0.01 |
| **Maternal age** | -.0001 | .0007 | 0.97 |
| **Maternal cigarette smoking during pregnancy** | -.0053 | .0102 | 0.60 |
| **Child sex** | .0030 | .0061 | 0.63 |
| **Birthweight** | -<.0001 | <.0001 | 0.44 |

**Supplementary Table 4.** Difference in DNA methylation levels between neonates born to anxious versus non-anxious mothers, stratified by infant sex.

|  | **Male infant** | | | **Female infant** | | |  |
| --- | --- | --- | --- | --- | --- | --- | --- |
| **CpG unit** | **∆** | **95% CI** | **p** | **∆** | **95% CI** | **p** | |
| ***IGF2* DMR0** |  |  |  |  |  |  | |
| 3 | -3.55% | -5.65 to -1.46% | 0.0010 | 0.08% | -2.23 to 2.39% | 0.95 | |
| 4 | 2.39% | -0.11 to 4.89% | 0.061 | -1.91% | -5.27 to 1.46% | 0.27 | |
| 6.7 | 0.09% | -1.52 to 1.70% | 0.91 | -0.38% | -2.34 to 1.58% | 0.70 | |
| Average | -0.22% | -1.95 to 1.51% | 0.81 | -1.01% | -2.94 to 0.92% | 0.30 | |
|  |  |  |  |  |  |  | |
| ***IGF2*/*H19* ICR** |  |  |  |  |  |  | |
| 1 | -1.77% | -3.95 to 0.41% | 0.11 | -3.34% | -5.84 to -0.83% | 0.0094 | |
| 5.6.7.8 | -0.74% | -2.87 to 1.40% | 0.49 | -3.36% | -5.73 to -0.99% | 0.0056 | |
| 11.12 | -1.63% | -3.78 to 0.52% | 0.13 | -2.71% | -4.91 to -0.51% | 0.016 | |
| 13.14 | -1.67% | -3.87 to 0.54% | 0.14 | -3.23% | -5.51 to -0.94% | 0.0059 | |
| 21.22 | -0.78% | -3.42 to 1.85% | 0.56 | -2.95% | -5.67 to -0.24% | 0.033 | |
| 23 | -1.07% | -3.81 to 1.66% | 0.44 | -2.68% | -6.00 to 0.62% | 0.11 | |
| Average | -1.04% | -2.99 to 0.92% | 0.29 | -3.70% | -5.90 to -1.51% | 0.0010 | |
